# Risk factors for early readmission to hospital in patients with malignancy-related ascites: a retrospective cohort study

**DOI:** 10.3389/fonc.2024.1409411

**Published:** 2024-10-01

**Authors:** Zhenhua Tian, Zhilong Huang, Yaqi Guo, Xiaolin Zhao, Luna Liu, Chunxiao Yu, Qingbo Guan

**Affiliations:** ^1^ Department of Endocrinology, Shandong Provincial Hospital, Shandong University, Jinan, China; ^2^ Key Laboratory of Endocrine Glucose & Lipids Metabolism and Brain Aging (Shandong First Medical University), Ministry of Education, Shandong Provincial Hospital Affiliated to Shandong First Medical University, Jinan, China; ^3^ Department of Endocrinology, Shandong Clinical Research Center of Diabetes and Metabolic Diseases, Jinan, China; ^4^ Department of Endocrinology, Shandong Institute of Endocrine and Metabolic Diseases, Jinan, China; ^5^ Shandong Engineering Laboratory of Prevention and Control for Endocrine and Metabolic Diseases, Jinan, China; ^6^ Department of Urology, Shandong Provincial Hospital, Shandong University, Jinan, China

**Keywords:** malignancy-related ascites, readmission, nationwide readmissions database, malignancy, ascites

## Abstract

**Introduction:**

Malignancy-related ascites (MRA) is a common serious complication of many advanced malignant tumors with high morbidity and mortality. The high hospital expenditures induced by unplanned readmission in patients with MRA have become an urgent issue to the public. We aimed to overall assess the unplanned early readmission rate of patients with MRA and explore the potential risk factors for such readmission.

**Methods:**

A retrospective cohort study based on 2018 Nationwide Readmissions Database was performed and patients with MRA were recruited into the analysis. The primary outcome was unplanned 30-day readmission rate and inpatient outcomes. The multivariate logistic regression analysis was performed to evaluate the potential risk factors for such early readmission.

**Results:**

Data obtained from 32,457 patients with MRA were analyzed, and of these 7,799 individuals (24.03%) were unplanned readmitted within 30-day follow-up. The mortality rate in the readmitted population was 15.15%. Patients at younger age were at a higher risk of readmission. The morbidities including hypertension (OR=1.117, 95%CI: 1.054-1.184), hyperlipemia (OR=1.075, 95%CI: 1.009-1.146) and diabetes (OR=1.118, 95%CI: 1.053-1.188), gastrointestinal malignancies and peritoneal procedure significantly increased the risk of 30-day readmission in patients with MRA.

**Discussion:**

More than one in five patients with MRA was unplanned readmitted within 30-day follow-up. The above risk factors should be timely intervened and the corresponding medical care should be strengthened in patients with MRA to lessen the unplanned readmission and improve the readmission outcomes.

## Introduction

Hospital readmission is a common, costly, and sometimes life-threatening event for hospitalized patients. It has been reported that almost one fifth of Medicare beneficiaries were readmitted within 30 days and the cost of unplanned rehospitalizations was up to $17 billion (1). To lower the hospital readmission rate and rehospitalization cost, the Centers for Medicare and Medicaid Services (CMS) has implemented several program initiatives including the Hospital Readmissions Reduction Program (HRRP) (2, 3), and the Hospital Value-Based Purchasing (HVBP) Mortality Program (4). Although some readmissions is unavoidable due to the disease progression, timely and qualifi ed medical care is important for avoidable readmissions (5). Therefore, identifying potential risk factors for hospital readmission and providing timely intervention is important.

Malignancy-related ascites (MRA) is a common complication of many advanced cancers, primarily caused by peritoneal carcinomatosis and occasionally due to primary tumors of peritoneum (6, 7). Gastrointestinal and gynecological cancers such as pancreas, colorectum, ovarian and uterine, are the most common causes of malignancy-related ascites (8, 9). These malignancies account for 70% of all MRA (10), and some reports has indicated that in up to 20% of malignant effusions, the primary origin of tumor is not identified (11). The presence of MRA accompanied by insufferable symptoms such as pain, nausea and vomiting, anorexia, difficulty in breathing and others, implies an advanced stage, poor prognosis and eventually a decreased survival rate (12–14). The complicated symptoms and persistent progression of disease contributes to the increased readmission rate, hospitalized cost and elevated mortality (15, 16). The goals of treatment for MRA are to relieve symptoms and reduce readmission and mortality. Thus, the overall assessment of readmission of MRA, and the identification of potential risk factor for readmission of MRA would be a substantial benefit to public health.

Data concerning the prevalence, economic burden and risk factors for readmission of MRA is scant and incomprehensive. Previous retrospective studies based on a limited number of patients have preliminarily explored the risk factors for the hospital length of stay and in-hospital mortality (17, 18). The small sample size of single-center studied population provides insufficient evidence for the verification of risk factors and makes it difficult to investigate the prevalence and economic burden of MRA. Recently, it has been published that 7 out of 2000 of out-patient were identified as MRA in National Inpatient Sample (NIS) data and MRA accounted for a total annual hospitalization cost of $6.4 billion (19). Ramamoorthy et al. have also showed that demographic, socioeconomic, and geographic factors were associated with inpatient mortality and hospital length of stay. However, this cross-sectional study did not assess the readmission rate and explore the risk factors for the readmission and hospital bad outcomes in patients with MRA, meanwhile it did not evaluate the effect of paracentesis on MRA, which is the most common diagnostic and therapeutic procedure in patients with MRA. Therefore, a large national retrospective cohort data-base analysis is needed to overall assess the readmission rate in patients with MRA and explore the risk factors for readmission.

In the study, we aimed to evaluate the national estimate of unplanned, all-cause 30-day readmission rates in patients with malignancy-related ascites using the Nationwide Readmissions Database (NRD), and to explore the potential risk factors associated with such readmissions.

## Patients and methods

### Data sources

The study population was drawn from the Nationwide Readmissions Database (NRD) 2018 sample (http://www.hcup-us.ahrq.gov/). The NRD as a nationally representative longitudinal database is constructed from Healthcare Cost and Utilization Project (HCUP) State Inpatient Databases (SID) that contains reliable, verified patient linkage numbers which can be used to track a patient across hospitals within a state. The 2018 NRD is derived from 28 geographically dispersed state, accounting for 59.7 percent of all American resident population and 58.7 percent of total hospitalized individuals. After restrict exclusions such as rehabilitation, long-term acute care hospitalizations, missing or questionable patient linkage numbers and discharged patients younger than 14 years old, the 2018 NRD consists of about 84 percent of all discharges from participating states. The database captures clinical and non-clinical variables using International Classification of Diseases-10^th^ revision (ICD-10) codes. Based on this database, we conducted a retrospective cohort study to evaluate unplanned, all-cause 30-day readmission rates for patients with MRA and to explore the potential risk factors associated with such readmission. The study was based on STROBE reporting guidelines and complied with the United States Agency for Healthcare Research and Quality’s Healthcare Cost and Utilization Project Data Use Agreement and was exempt from research ethics board review. Data from the NRD 2018 was de-identified; therefore, consent to participate was not applicable. We previously have mentioned such information in the Declarations part, and we supplied the information in the Methods section according to the suggestion.

### Study population

**Figure 1:**
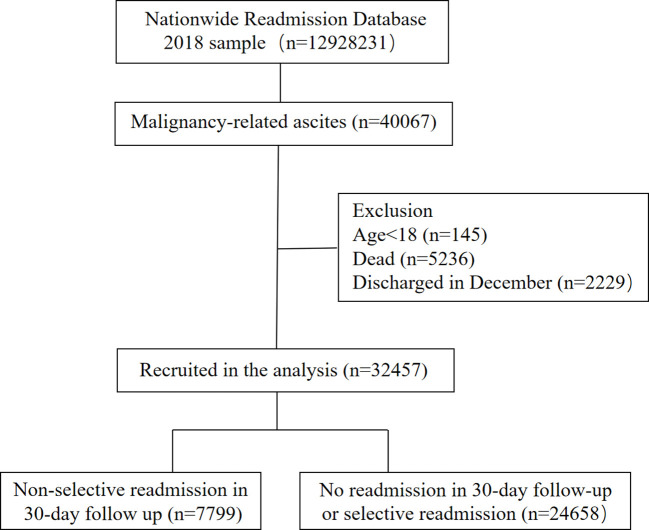
Flowchart of the analysis.

We included patients with a first discharged diagnosis of malignancy-related ascites (n=40,067) as defined by ICD-10 codes. The malignancy-related ascites was diagnosed as: (1) malignant ascites as primary diagnosis; (2) malignant ascites as secondary diagnosis; (3) other unspecified ascites as primary diagnosis and malignancies as secondary diagnosis; (4) malignancies as primary diagnosis and other unspecified ascites as secondary ascites. Patients who were younger than 18 years old (n=145) were excluded from our study. Given that the patient linkage numbers can’t be tracked across years in NRD database, we excluded patients who were first discharged during December (n=2229) and patients died during the first admission (n=5236) to evaluate the 30-day readmission rates of patients with MRA. Eventually, a total of 32457 patients with discharged diagnosis of MRA were included to the final analysis to observe the early readmission rate and hospitalized outcomes (**Figure 1**).

### Outcomes

The primary outcome was early unplanned readmission within 30 days period after first hospitalization, and the only first readmission was recruited to the analysis. In addition, the readmission was defined as all-cause readmission, elective readmission and transferred hospitalization was not considered as readmission. The secondary outcomes included total charges of hospitalization, hospital length of stay and inpatient mortality. Meanwhile, the reasons for readmission were recruited into the analysis.

### Data collection

For each patient, we collected the following factors: age, sex, median household income for patient’s ZIP code, expected primary payer (Medicare, Medicaid, private insurance, self-pay, no charge and other), patient location (“central” counties of metro areas of ≥1 million population, “fringe” counties of metro areas of ≥1 million population, counties in metro areas of 50,000-249,999 population, counties in metro areas of 50,000-249,999 population, micropolitan counties, not metropolitan or micropolitan counties), comorbidities (obesity, hypertension, hyperlipemia, diabetes, chronic kidney disease, heart failure), the all patient refined DRGs (APRDRG) risk mortality (minor likelihood of dying, moderate likelihood of dying, major likelihood of dying, extreme likelihood of dying), APRDRG severity (minor loss of function includes cases with no comorbidity or complication, moderate loss of function, major loss of function, extreme loss of function). The primary malignancy type (liver, pancreas, colon and rectums, other gastrointestinal tract, ovary, corpus and uterus, male genital system, urinary, hematologic system, others) and procedure type (diagnostic peritoneal paracentesis only, peritoneal drainage, peritoneal drainage with drainage devices) were captured.

### Statistical analysis

Categorical variables were described as proportions and continuous variables were presented as mean ± standard variations (SD). We used Chi-squared test and independent two-sample t-test to analyze between-group differences of categorical variables and continuous variables respectively. In addition, the readmission rates were presented as percentages on day 1 to 30 after first hospitalization and different primary malignancy types were described as proportion in the population with MRA. Multivariate logistic regression analysis was performed to evaluate the potential risk factors for readmission. The models were adjusted for several covariates, including age, sex, primary expected payer, patient location, APRDRG risk mortality, APRDRG severity, comorbidities, primary malignancy type, and procedure type. All statistical analyses were performed using SPSS statistical software (version22.0) and a two-sided p value lower than 0.05 were considered as statistically significant.

## Results

### Baseline clinical characteristics of patients with MRA and 30-day readmission rates

**Figure 2:**
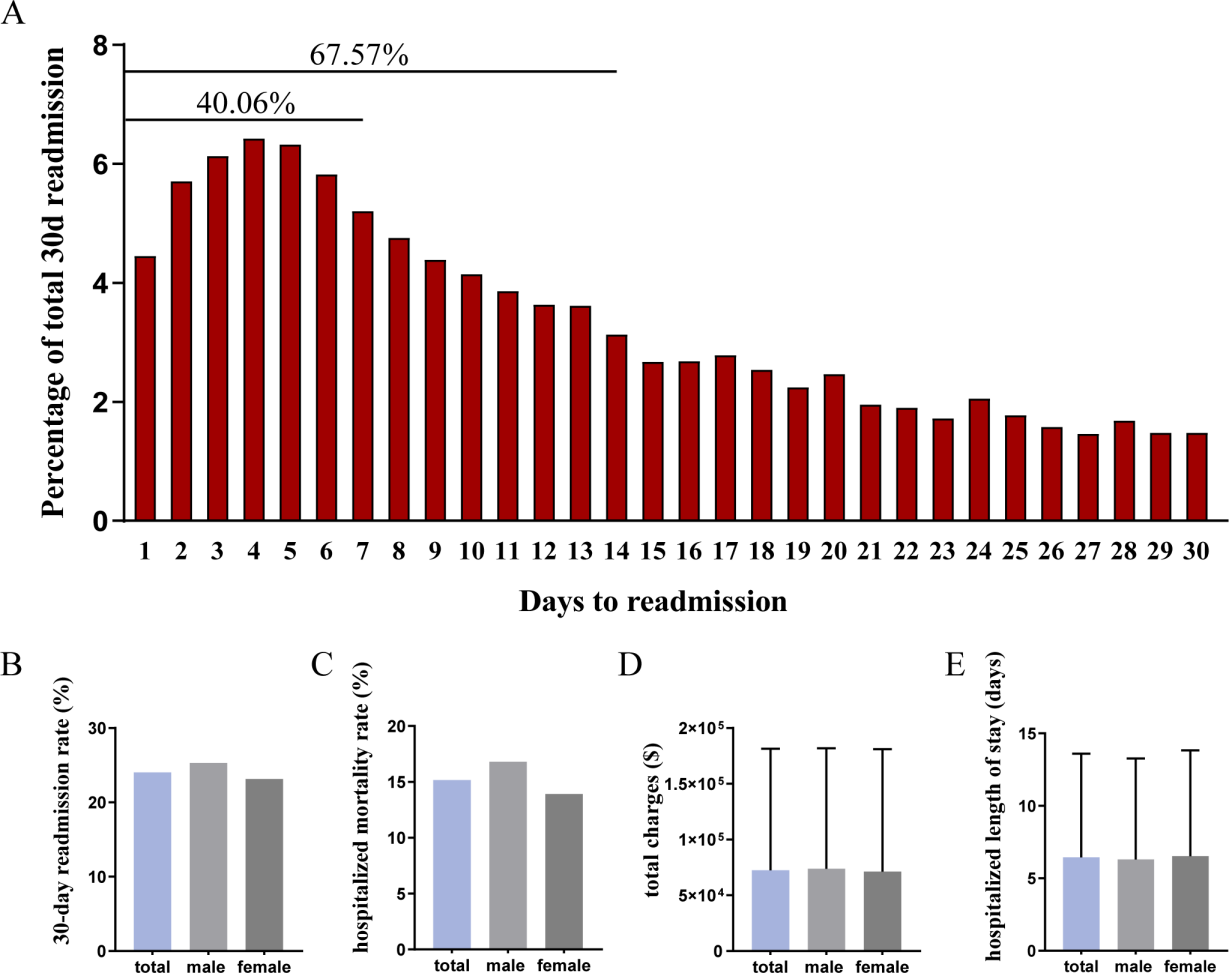
Hospitalized outcomes in the readmitted patients with MRA. **(A)** cumulative readmission rates in the 30-day follow-up; **(B)** unplanned 30-day readmission rate; **(C)** inpatient mortality rate; **(D)** hospitalized total charge; **(E)** hospitalized length of stay.

In the study, a total of 32,457 patients with MRA were recruited to the study, and of these 7,799 individuals (24.03%) were unplanned readmitted within 30-day follow-up (**Table 1**, **Figure 2B**). In the whole studied cohort, 70.28% of participants were older than 60 years old, and 59.38% were females (**Table 1**). Of these 55.25% patients were paid by Medicare, and 28.56% have private insurance. More than half of patients (58.82%) were located in central and fringe counties and a great number of patients were subclassed as major likelihood of dying (59.57%) and major loss of function (56.50%) according to the all patient refined DRGs, which are assigned using software developed by 3M Health Information Systems. A total of 41.47% patients were treated with a peritoneal procedure, of which diagnostic peritoneal paracentesis only accounted for 9.38%, peritoneal drainage accounted for 26.23%, and peritoneal drainage with drainage devices accounted for 5.87% (**Table 1**). Compared to non-readmitted and selective readmitted patients, those with 30-day readmission had significantly different age distribution (*P*<.001), sex proportion (*P*<.001), and discrepant percentage of expected primary payer (*P*<.001) and patient location (*P*<.001) (**Table 1**). Patients with early readmission had a higher proportion of hypertension (*P*<.001), hyperlipemia (*P*=0.029), diabetes (*P*<.001), chronic kidney disease (*P*=0.018) and peritoneal procedures (*P*<.001). No significant difference of household income, rates of obesity (*P*=0.050) and heart failure (*P*=0.757) was observed between studied patients with readmission and those without readmission (**Table 1**).

**Table 1:** Baseline characteristics of hospitalizations for malignancy-related ascites.

	Total (n=32457)	Without readmission (n=24658)	With readmission (n=7799)	*P*
Age by category (%)				<.001
18-44	5.97	5.71	6.80	
45-59	23.75	23.23	25.40	
60-74	45.40	44.62	47.88	
≥75	24.88	26.45	19.93	
Female (%)	59.38	60.07	57.19	<.001
House income (%)				0.573
1. 0-25th percentile ($1 - $45,999)	22.91	22.82	23.22	
2. 26th to 50th percentile ($46,000 - $58,999)	25.91	25.94	25.82	
3. 51st to 75th percentile ($59,000 - $78,999)	26.05	26.22	25.51	
4. 76th to 100th percentile ($79,000 or more)	25.12	25.02	25.45	
Expected primary payer (%)				<.001
1. Medicare	55.25	56.01	52.84	
2. Medicaid	11.72	11.11	13.64	
3. Private insurance	28.56	28.35	29.24	
4. Self-pay	2.01	2.02	1.98	
5. No charge	0.27	0.27	0.26	
6. other	2.20	2.24	2.05	
Patient location (%)				<.001
1.”central” counties of metro areas of ≥1 million population	31.70	30.78	34.60	
2.”fringe” counties of metro areas of ≥1 million population	27.52	27.44	27.76	
3.counties in metro areas of 250,000- 999,999 population	19.97	20.07	19.63	
4.counties in metro areas of 50,000- 249,999 population	8.42	8.67	7.64	
5.micropolitan counties	6.99	7.33	5.89	
6.not metropolitan or micropolitan counties	5.41	5.70	4.48	
APRDRG risk mortality (%)				<.001
1. minor likelihood of dying	2.11	2.35	1.35	
2. moderate likelihood of dying	15.42	15.84	14.10	
3. major likelihood of dying	59.57	58.21	63.84	
4. extreme likelihood of dying	22.90	23.59	20.71	
APRDRG severity (%)				<.001
1. minor loss of function (includes cases with no comorbidity or complication)	0.21	0.24	0.14	
2. moderate loss of function	14.54	15.21	12.41	
3. major loss of function	56.50	55.39	59.99	
4. extreme loss of function	28.75	29.15	27.45	
Comorbidities				
Obesity (%)	10.44	10.25	11.03	0.050
Hypertension (%)	53.98	53.43	55.71	<.001
Hyperlipemia (%)	24.61	24.32	25.54	0.029
Diabetes (%)	28.44	27.59	31.11	<.001
Chronic kidney disease (%)	14.54	14.28	15.36	0.018
Heart failure(%)	10.57	10.54	10.67	0.757
Procedure type (%)				<.001
Diagnostic peritoneal paracentesis only	9.38	8.92	10.82	
Peritoneal drainage	26.23	24.73	30.97	
Peritoneal drainage with drainage devices	5.87	5.94	5.65	
No procedure	58.53	60.42	52.56	

P, patients with readmission vs patients without readmission

### Common types of malignancies in patients with MRA

**Figure 3:**
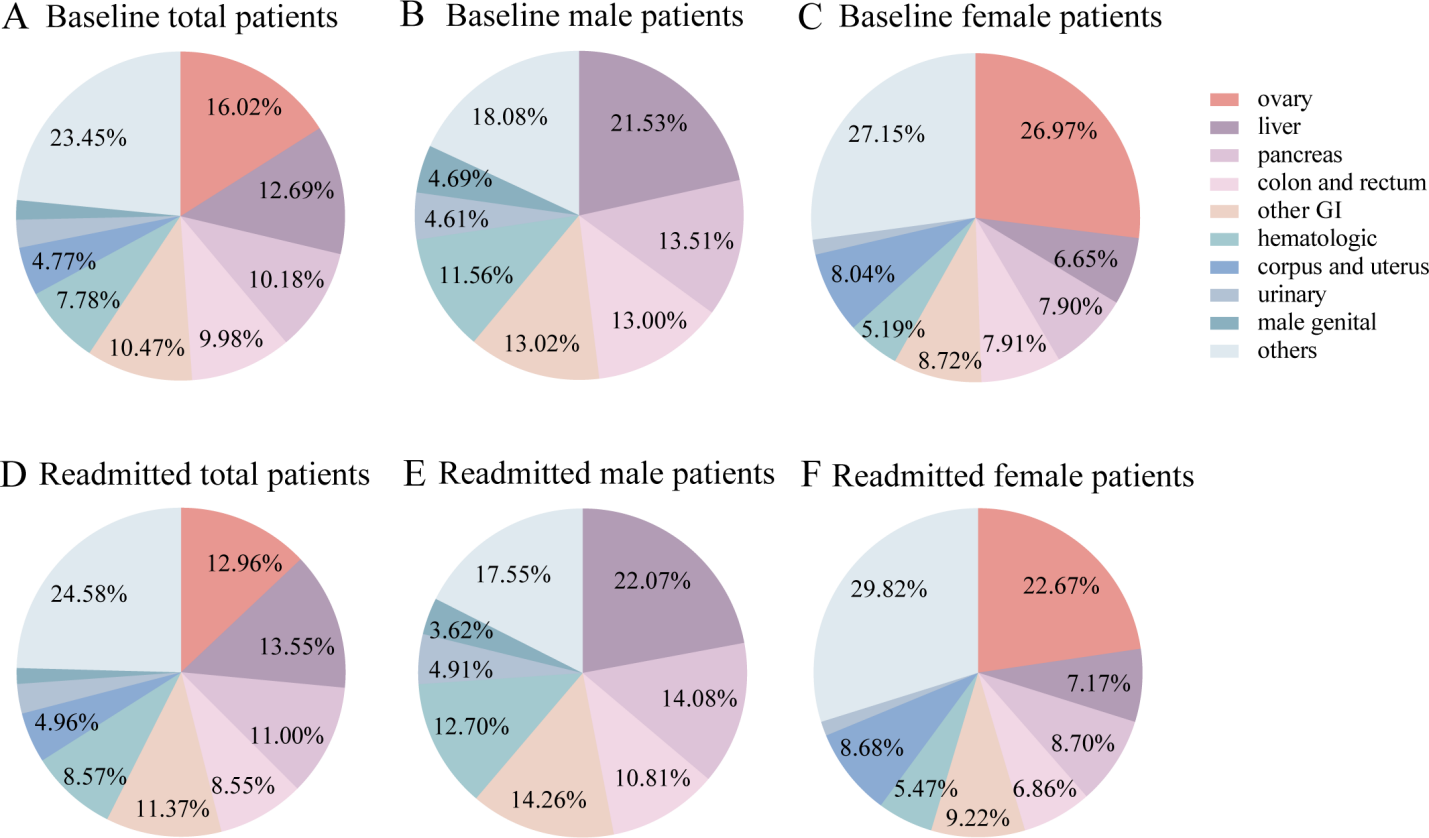
Primary malignancy type in patients with MRA. **(A)** baseline whole patients; **(B)** baseline male patients; **(C)** baseline female patients; **(D)** readmitted patients; **(E)** readmitted male patients; **(F)** readmitted female patients.

To evaluate the proportion of different primary malignancies in patients with MRA, we calculated the percentage of different primary malignancies in whole, male and female cohort respectively due to the different incidence of cancer between genders. In the whole studied cohort, the most common cancer was ovarian (16.02%), followed by liver and intrahepatic bile duct (12.69%), other gastrointestinal tract (10.47%), pancreas (10.18%), colon and rectum (9.98%), hemopoietic system (7.78%), corpus and uterus (4.77%), urinary system (2.75%), male genital system (1.91%) and others (23.45%) (**Figure 3A**). Correspondingly, liver and intrahepatic bile duct malignancies became the most common cancer in male patients with MRA and accounted for 21.53%, followed by pancreas (13.51%), other gastrointestinal tract cancers (13.02%) and colon and rectum (13.00%) (**Figure 3B**). The most common malignancies in the female cohort were ovary (26.97%), other gastrointestinal tract (8.72%), corpus and uterus (8.04%), pancreas (7.90%), colon and rectum (7.91%), liver and intrahepatic bile duct (6.65%), hemopoietic system (5.19%), urinary system (1.47%) and others (27.15%) (**Figure 3C**). To compare the effect of primary malignant type on earlier readmission, we calculated the proportion of cancer type in unplanned 30-day readmitted patients. Importantly, the percentage of liver and intrahepatic bile duct cancer (13.55% vs 12.69%) and pancreatic cancer (11.00% vs 10.18%) was increased in unplanned readmitted patients in comparison to the whole baseline cohort, meanwhile there was a decreased tendency for the proportion of ovary cancer in the unplanned readmitted patients (12.96% vs 16.02%) (**Figure 3D**). The subgroup analysis of male and female population showed a consistent fluctuating trend of proportion of primary malignancies (**Figure 3E** vs **Figure 3B**, **Figure 3F** vs **Figure 3C**). Given the different proportion of malignancies in the patients with MRA, we performed sensitivity analysis in patients with gastrointestinal malignancy-related ascites and patients with genital-malignancy related ascites respectively. The detailed results about risk factors for unplanned readmission in above mentioned subgroups were shown in **Supplementary Figures S2**, **S3** respectively.

### Burden and cost of readmission in patients with MRA

To observe the distribution of readmission interval time in patients with MRA, we figured up the cumulative readmission rate during a follow-up period of 30 days. It showed that the readmission rate was remained at a high level in the first week, and accounted for a cumulative 40.06% of readmission rates (**Figure 2A**). In the second week, the readmission rate gradually and slowly declined and lead to a total of 27.51% of readmission rates, then the readmission rate maintained at a relatively constant low level (**Figure 2A**). We also showed that the mortality rate in the readmitted population was 15.15% (**Figure 2C**), the mean of total charges during hospitalization was 72383$ (**Figure 2D**), and the mean of hospitalized length of stay was 6.44 days (**Figure 2E**). In addition, we observed that the readmission rate (25.32 vs 23.14%, *P*<.001) and hospitalized mortality rate (16.81% vs 13.90%, *P*<.001) in male readmitted patients was higher than female patients, however, no significant difference of the total charges (73824 ± 108082 vs 71304 ± 109760, *P*=0.313) and hospitalized length of stay (6.31 ± 6.96 vs 6.53 ± 7.30, *P*=0.183) existed between male and female cohort (**Figure 2B–E**). Meanwhile, we observed the reasons for readmission in the patients with MRA. As it was shown in **Supplementary Figure S1**, sepsis accounted for 13.68%, followed by secondary malignancy of peritoneum (7.22%), ovary malignancy (3.32%), pancreas malignancy (2.85%), hepatic failure (2.78%) and liver and intrahepatic bile duct malignancy (2.67%).

### Risk factors for 30-day readmission in patients with MRA

**Figure 4:**
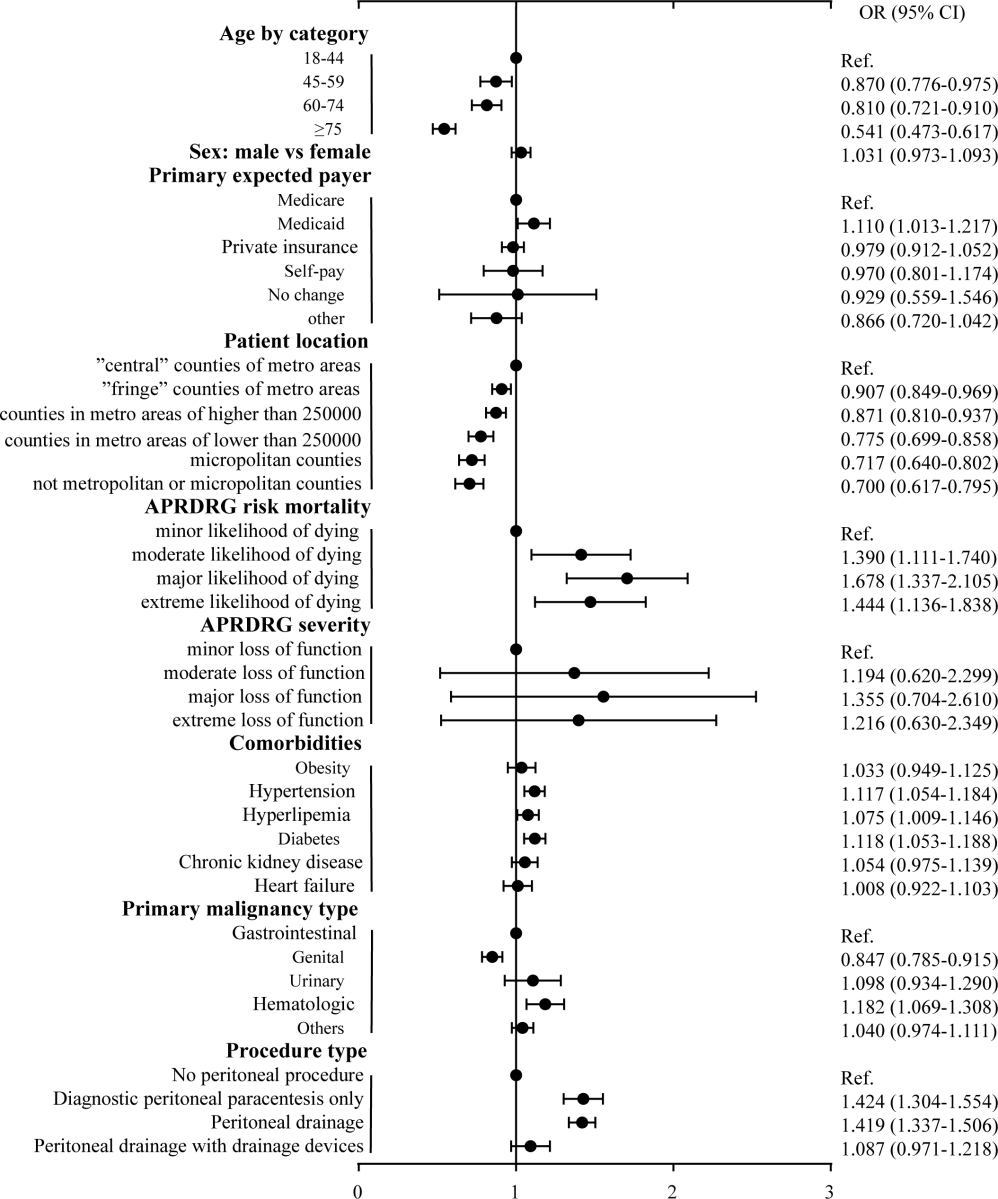
Multivariable analysis of factors associated with 30-day readmission to hospital for malignancy-related ascites. OR, Odds ratio; CI, confidence interval; Ref, reference.

To investigate the risk factors for 30-day readmission, we performed Multiple Logistic Regression analysis. The results showed that older age significantly decreased the risk of unplanned 30-day readmission compared to the young patients, especially in the patients older than 75 years old, the risk of readmission decreased to 0.541-fold (95% CI: 0.473-0.617, *P*<.001) (**Figure 4**). Patients paid by Medicaid had a significant higher risk of readmission than patients paid by Medicare (OR=1.110, 95%CI: 1.013-1.217, *P*=0.025). Male patients had a higher risk of 30-day readmission than female patients, but the association remained not significant (OR=1.031, 95%CI: 0.973-1.093, *P*=0.306). The classifications of primary expected payer and APRDRG severity were non-significantly associated with the risk of 30-day readmission (*P*>0.05). Patients in the smaller counties had a significantly lower risk of readmission compared to patients in the large counties (*P*<0.05). The odds ratios for 30-day readmission in patients with moderate likelihood of dying, major likelihood of dying and extreme likelihood of dying in contrast to patients with minor likelihood of dying were 1.390 (95%CI: 1.111-1.740, p=0.004), 1.678 (95%CI: 1.337-2.105, *P*<.001) and 1.444 (95%CI: 1.136-1.838, *P*=0.003) respectively (**Figure 4**). Patients with hypertension had a higher risk of 30-day readmission than patients without hypertension (OR=1.117, 95%CI: 1.054-1.184, *P*<.001), meanwhile hyperlipemia (OR=1.075, 95%CI: 1.009-1.146, *P*=0.026) and diabetes (OR=1.118, 95%CI: 1.053-1.188, *P*<.001) increased the risk of 30-day readmission (**Figure 4**). The risk of 30-day readmission in patients with genital malignancies related ascites decreased to 0.847-fold (95% CI: 0.785-0.915, *P*<.001) in comparison to patients with gastrointestinal malignancies, and hematologic malignancy related ascites increased the risk of readmission (OR=1.182, 95%CI: 1.069-1.308, *P*<.001), while urinary and others malignancy did not significantly affect the 30-day readmission (*P*>0.05). Compared to patients without peritoneal procedure, the risk of 30-day readmission in patients with diagnostic peritoneal paracentesis only and patients with peritoneal drainage increased to 1.424 (95%CI: 1.304-1.554, *P*<.001) and 1.419 (95%CI: 1.337-1.506, *P*<.001) respectively, while patients which were treated with peritoneal drainage with drainage devices did not have an increased risk of 30-day readmission (OR=1.087, 95%CI: 0.971-1.218, *P*=0.148) (**Figure 4**). We further performed sensitivity analysis in patients clearly diagnosed with malignant ascites (**Supplementary Figure S2**). The risk factors for unplanned 30-day readmission were considerably consistent with the whole cohort diagnosed with MRA. However, male patients had a significant higher risk of admission than female patients (OR=1.153, 95%CI: 1.033-1.287, *P*=0.011), and hyperlipemia (OR=1.091, 95%CI: 0.977-1.218, *P*=0.122) was not independent risk factor for readmission, and malignancies type did not affect the risk of unplanned readmission in the sensitivity analysis (**Supplementary Figures S3**, **S4**).

## Discussion

Malignancies-related ascites remains a prominent chronic disease globally, characterised by its high morbidity and mortality. Hospital expenditures exacerbated by MRA-associated unplanned readmissions and accompanying adverse outcomes have become an increasingly important issue. With the clinical, financial and administrative burden continuing to put pressure on healthcare providers worldwide, it is increasingly important to evaluate the early unplanned readmission rates among MRA patients and explore the potential risk factors driving these readmissions. In this study, we found that 24.03% of individuals with MRA were unplanned readmitted within 30 days and that the majority of MRA incidences derived from gastrointestinal and genital cancer. It is demonstrated herein that young age, being paid by Medicaid, being located in large counties, morbidities including hypertension, hyperlipemia and diabetes, gastrointestinal malignancies and peritoneal procedure all significantly increased the risk of 30-day unplanned readmission among MRA patients. Based on these novel findings, implementing timely and targeted interventions offers promise in making the allocation of health care resources more efficient, as well as improving MRA patient outcomes.

Our findings revealed a significantly different sex distribution in readmitted MRA patients. Prior to this study, Ramamoorthy et al. have shown that males were associated with higher inpatient mortality and longer hospitalisations (length of stay), which was consistent with our study (19). We infer that the varying types of tumors prone to affect each sex explain much of the sex difference. There was a significantly higher proportion of genital cancer and a relatively lower proportion of gastrointestinal cancer among female MRA patients relative to male patients. Simultaneously, we found that the gastrointestinal cancer independently and significantly increased the probability of the 30-day readmission rates relative to genital cancer in our studied cohort. Previous studies have shown that ovarian cancer prompted longer survival whereas liver metastases adversely affected survival (18). Apart from malignancy types, different chronic complications, immune and metabolic statuses between males and females are also likely to contribute to sex difference regarding early readmission and inpatient mortality rates. Although the pertinent mechanisms have not yet been elucidated, this study provided preliminary evidence for the relatively lower readmission and better hospitalized outcomes among female MRA patients. Consequently, patients with MRA, especially males, should receive careful and sustained clinical care to evaluate the risk of ascites recurrence and reduce readmission rates.

A higher readmission rate in young patients (18-44 years old) was observed in this study, relative to older MRA patients, which is in accordance with previous studies utilising NRD analysis (20, 21). Therein, it has been reported that younger patients undergoing surgery related to ovarian cancer or hepato-pancreatic surgery exhibited an increased risk of readmission compared to older patients (20, 21). Although the exact mechanisms have not been clarified, previous study has indicated that men with advanced prostate cancer who were diagnosed at a young age exhibit a higher mortality than men diagnosed at an older age (22). Young age was also found to be modestly associated with poorer progression-free survival among patients with colorectal cancer (23). These observations align with previous studies that have highlighted the impact of young age on patient outcomes. Based on these prior findings, we speculated herein that tumors were more likely to progress and worsen in young patients with malignancy, a phenomenon which ultimately contributed to the distinction of readmission rates between younger and older patients. Another explanation could be that older patients may have a higher risk of outpatient mortality due to an increased probability of multiple comorbidities, which can contribute to the underestimation of readmission rates (24). Consequently, and contrary to the stereotype of strengthening care for the elderly, this study found that it was young MRA patients who should be given more focused clinical attention. This important finding can guide clinical nursing practice and thus reduce readmission rates.

Apart from demographic factors including sex and age, this study also found that socioeconomic, and geographic factors influenced hospitalized outcomes. We showed that patient location was associated with the risk of 30-day readmission, and being located in major counties increased the risk of early readmission, which was not accordance with previous research (19). However, this phenomenon reflects at least in part that patients in minor counties may have less access to high-quality medical resources. Moreover, patients who paid for their healthcare via Medicaid demonstrated a higher risk of readmission than those paid by Medicare. Conversely, the household income and other categorisations of primary payer were not found to be associated with early readmission risk among MRA patients. These results indicated that other complicating factors, such as social services and hospital resources, are likely to have an important association, and further study into these factors is needed.

This research found that comorbidities including hypertension, hyperlipemia or diabetes considerably increased the risk of 30-day readmission among MRA patients, while obesity, chronic kidney disease and heart failure demonstrated no strong association with readmission risk. Comorbidities have already been found to increase the risk of readmission in patients with malignant cancer (25–28), however, it remains uncertain whether these comorbidities directly contribute to the progression of malignancies or merely increase readmission rates due to the corresponding conditions. Thus, findings herein suggested that patients should be treated for metabolic disorders including blood pressure, serum glucose and lipids, so as to reduce the early readmission and adverse hospitalized outcomes regardless of the unspecified mechanism. Our study also demonstrated that patients categorized with a major or extreme likelihood of dying based on APRDRG risk mortality had an increased risk of readmission, compared to those assigned a minor likelihood of dying. The classification of APRDRG disease severity was not significantly associated with readmission risk. However, the lack of detailed information concerning disease severity of malignancies-related ascites in the NRD dataset may contribute to statistical biases in any analysis of disease severity and readmission rates. Notwithstanding, serum glucose, lipids and pressure should be routinely checked, with timely intervention ensuring these indexes stay within normal ranges. Doing so is likely to reduce the readmission rate and improve the readmission outcomes in patients with MRA.

Clinically, there is no standard treatment for malignancies-related ascites (29). Conventional methods in managing malignancies-related ascites including diuretics, sodium and fluid restriction and instillation of chemotherapeutic agents have proven to be unsuccessful (30), therefore, some peritoneal procedures were necessary to control for excessive fluid accumulation. Our findings demonstrated that diagnostic peritoneal paracentesis and peritoneal drainage without devices significantly increased the risk of 30-day readmission in MRA patients. High drainage volume of intraoperative ascites was was found to be an independent risk factor for postoperative complications among patients with malignant ascites (31). Nonetheless, we speculated that this result was biased due to the severity of malignancies-related ascites. We have made preliminary adjustments for subclasses of APRDRG severity when assessing the association of peritoneal procedure and malignancies-related ascites, yet despite this, the lack of record regarding the severity of ascites and other treatments including diuretics, sodium and fluid restriction ultimately limited the accuracy of the analysis. Meanwhile, we have demonstrated peritoneal drainage with devices did not increase the risk of early readmission among MRA patients. Previous studies have proposed specific peritoneal drainage methods including percutaneous peritoneovenous shunt and automated pump devices, thereby evaluating the safety and effectiveness in MRA treatment (32, 33). Further studies are needed to evaluate the strength of association and provide more definitive evidence to guide the clinical treatment of MRA. Meanwhile, patients who have undergone peritoneal procedures should be regularly assessed regarding the development, deterioration and recurrence of MRA. Our findings, whilst informative, ultimately highlight that considerably more research into other effective pharmacological interventions or aggressive approaches towards readmission prevention are urgently required.

### Limitations

Several limitations existed in this study. The reliance on ICD-10 codes may contribute to the inaccuracy of diagnosis. And given the low detection rate of malignant cells in ascites examination, we recruited patients with unspecified ascites with malignancies besides patients with malignant ascites into the final cohort analysis. To avoid the misclassification bias, we performed sensitivity analysis in the patients clearly diagnosed with malignant ascites and found that 26.17% of patients were unplanned readmitted in the follow-up period, which was similar with the whole cohort analysis. Besides, the lack of information about the severity of MRA, relevant medications and elaborate treatment in NRD led to attenuated estimates in our findings. Therefore, we investigated the effect of comorbidities and peritoneal procedure on readmission rate, meanwhile we added some indexes assessing health status including APRDRG risk mortality and severity in our analysis. Despite our efforts to control for known variables, it is important to acknowledge that there may be unmeasured confounding factors influencing the observed outcomes. Future studies should consider investigating these factors to provide a more comprehensive understanding of the complexities surrounding early readmission risks in MRA patients. We were incapable of capturing mortality data out of hospitals due to the characteristics of NRD database. Correspondingly, we analyzed the earlier 30-day readmission rate to minimize the impact of out-of-hospital mortality. Further study should be performed to recruit the out-of-hospital mortality into analysis and explore the risk factor for unplanned readmission in patients with MRA.

## Conclusions

In conclusion, our study reported that about one in five individuals with MRA were unplanned readmitted within 30 days, meanwhile young age, paid by Medicaid, major counties, morbidities including hypertension, hyperlipemia and diabetes, gastrointestinal malignancies, and peritoneal procedure significantly increased the risk of 30-day readmission in patients with MRA. The above risk factors should be timely intervened and the corresponding medical care should be strengthened in patients with MRA to lessen the unplanned readmission and improve the readmission outcomes. Future studies focused on the effect of corresponding intervention on early readmission rate in patients with MRA are needed to verify these risk factors and explore specific mechanisms.

### Clinical recommendations

Based on our findings, several actionable recommendations for clinical practice can be made to improve outcomes for patients with malignancy-related ascites (MRA). First, healthcare providers should implement targeted strategies for early identification and management of high-risk patients, such as those with gastrointestinal malignancies, young age, and significant comorbidities like hypertension and diabetes. Regular monitoring and proactive management of these risk factors can potentially reduce the likelihood of early readmission. Second, given the higher risk associated with peritoneal procedures, clinicians should carefully evaluate the necessity of such interventions and consider alternative management options where feasible. Furthermore, to address the socioeconomic and geographic disparities observed, tailored care plans that account for patients’ access to healthcare resources and financial coverage should be developed. Finally, continuous education and support for patients and their families regarding the management of chronic conditions and the importance of follow-up care can help mitigate the risk of readmission. By integrating these recommendations into clinical practice, healthcare professionals can enhance patient care and potentially reduce readmission rates among MRA patients.

## Data Availability

The data analyzed in this study was obtained from https://hcup-us.ahrq.gov/nrdoverview.jsp. The data can only be used for non-commercial research purposes. Requests to access these datasets should be directed to Tian Zhenhua, 13853188086@163.com.
