# Supplementary material for: Risk factors for early readmission to hospital in patients with malignancy-related ascites: a retrospective cohort study
Source: Front Oncol. 2024 Oct 1;14:1409411. doi: 10.3389/fonc.2024.1409411 (PMC11474032; doi:10.3389/fonc.2024.1409411)
Supplement: Supplementary file 1 [file DataSheet1.docx]

Risk Factors for Early Readmission to Hospital in Patients with Malignancy-related Ascites: A retrospective cohort study

Zhenhua Tian^1,^^2,3,4,5^, Zhilong Huang^6^, Yaqi Guo^2,3,4,5^, Xiaolin Zhao^2,3,4,5^, Luna Liu^1,2,3,4,5^, Chunxiao Yu^1,2,3,4,5,#^, Qingbo Guan^1,2,3,4,5,#^

| Supplementary Figure 1 | The Reason for readmission in patients with MRA. |
| --- | --- |
| Supplementary Figure 2 | Multivariable analysis of factors associated with 30-day readmission to hospital in patients with malignant ascites |
| Supplementary Figure 3 | Multivariable analysis of factors associated with 30-day readmission to hospital in patients with gastrointestinal malignancy-related ascites |
| Supplementary Figure 4 | Multivariable analysis of factors associated with 30-day readmission to hospital in patients with genital malignancy-related ascites |
| Supplementary Table 1 | The ICD-10 code in the analysis. |

Supplementary Figure 1 The Reason for readmission in patients with MRA.


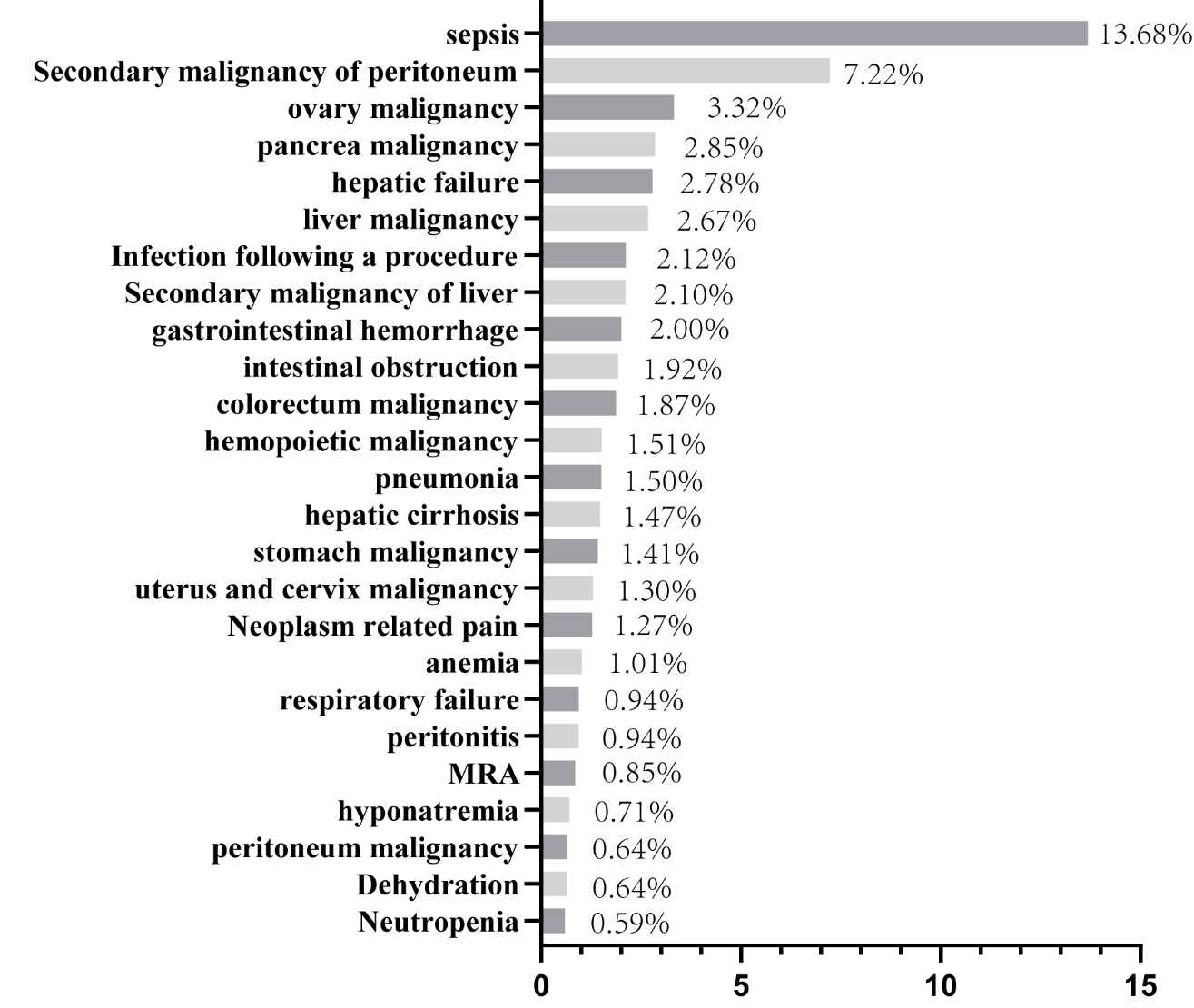


Supplementary Figure 2 Multivariable analysis of factors associated with 30-day readmission to hospital in patients with malignant ascites ^a^.


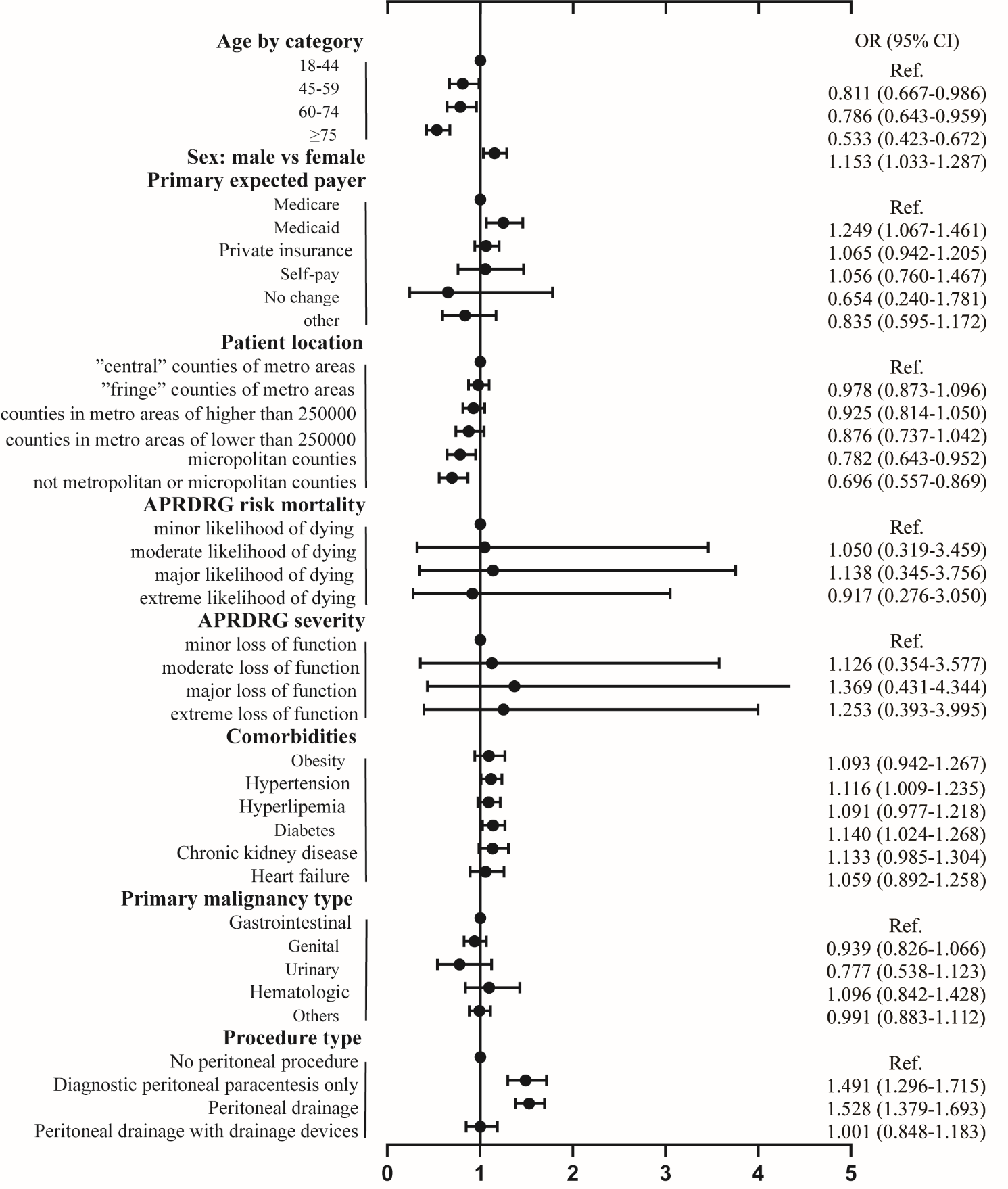


^a^ The sensitivity analysis in the patients clearly diagnosed with malignant ascites (the unplanned 30-day readmission rate was 26.17%). Abbreviations: OR: Odds ratio; CI: confidence interval; Ref: reference.

Supplementary Figure 3 Multivariable analysis of factors associated with 30-day readmission to hospital in patients with gastrointestinal malignancy-related ascites ^a^.


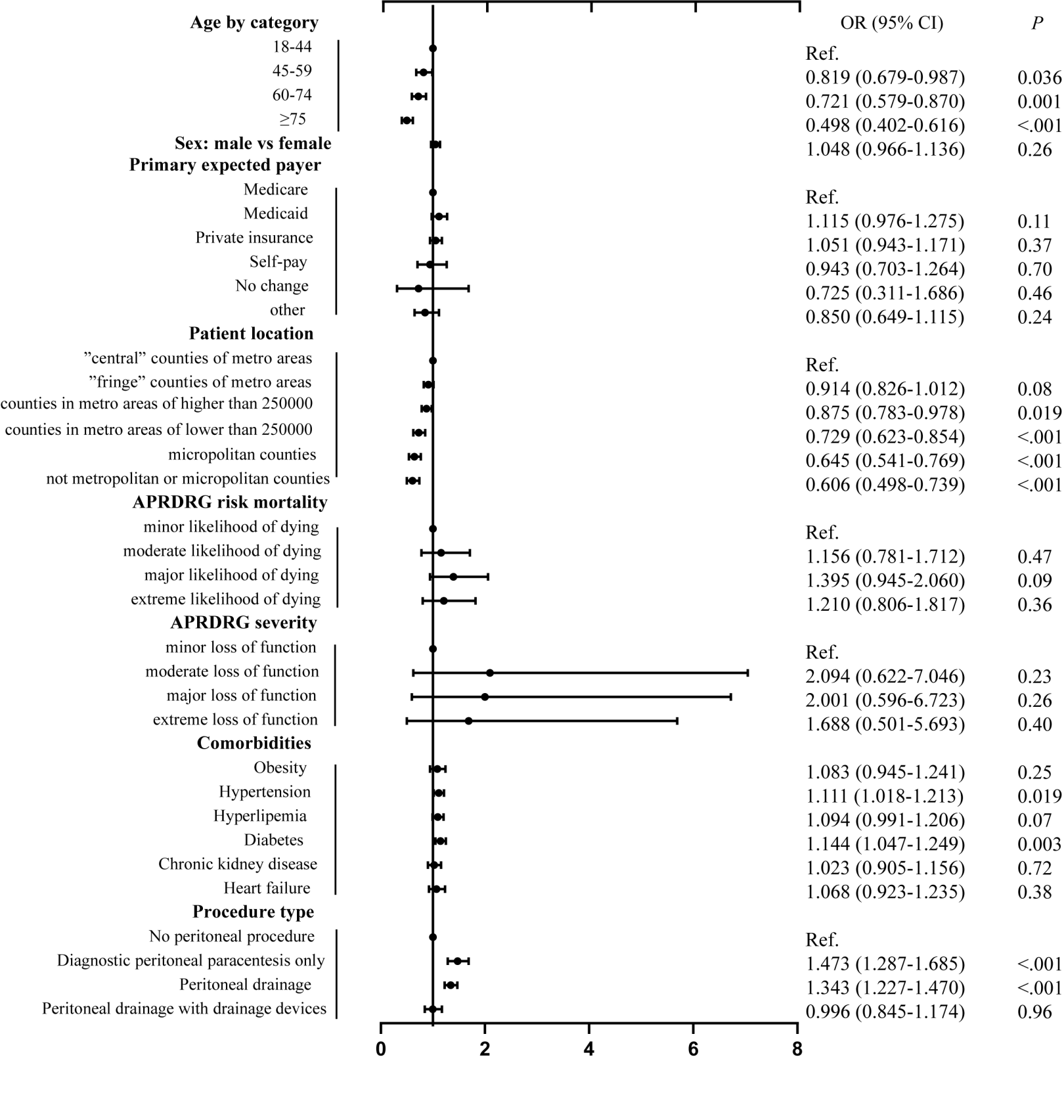


^a^ The sensitivity analysis in the patients clearly diagnosed with gastrointestinal malignancy-related ascites. Abbreviations: OR: Odds ratio; CI: confidence interval; Ref: reference.

Supplementary Figure 4 Multivariable analysis of factors associated with 30-day readmission to hospital in patients with genital malignancy-related ascites ^a^.


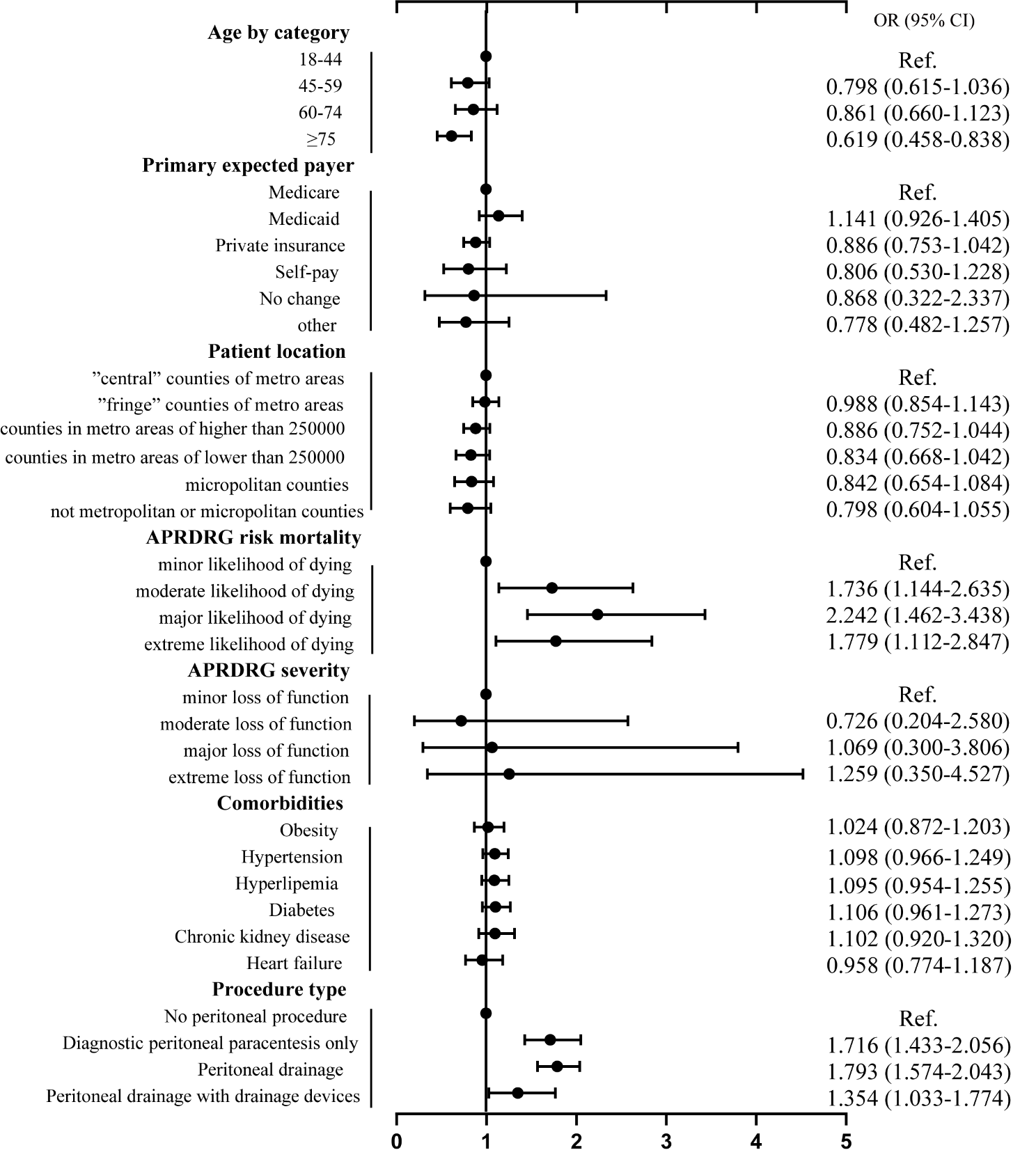


^a^ The sensitivity analysis in the patients clearly diagnosed with genital malignancy-related ascites. Abbreviations: OR: Odds ratio; CI: confidence interval; Ref: reference.

Supplement Table 1 The ICD-10 code in the analysis.

| Disease | code |
| --- | --- |
| Malignant ascites | R180 |
| Other ascites | R188 |
| Liver and intrahepatic bile duct cancer | C220, C221, C222, C223, C224, C227, C228 |
| Pancreas cancer | C250, C251, C252, C253, C254, C257, C258, C259 |
| Colon and rectum cancer | C180, C182, C183, C184, C185, C186, C187, C188, C189 |
| Other gastrointestinal cancer | C153, C154, C155, C158, C159, C160, C161, C162, C164, C165, C166, C168, C169, C170, C171, C172, C173, C178, C179, C210, C211, C212, C218,C229, C23, C240, C241, C248, C249,C260, C261, C269, C480, C481, C482, C488, C49A0, C49A1, C49A2, C49A3, C49A4, C49A5, C49A9 |
| Ovarian cancer | C561, C562, C569 |
| Corpus and uterus cancer | C530, C531, C538, C539,C540, C541, C542, C543, C548, C549,C55 |
| Other female genital cancers | C510, C511, C512, C518, C519, C52, C5700, C5701, C5702, C5710, C5711, C5712, C5720, C5721, C5722, C573, C574, C577, C578, C579, C58 |
| Male genital cancer | C61, C6200, C6201, C6202, C6210, C6211, C6212, C6290, C6291, C6292, C600, C601, C602, C608, C609, C6300, C6301, C6302, C6310, C6311, C6312, C632, C637, C638 |
| Urinary cancer | C670, C671, C672, C673, C674, C675, C676, C677, C678, C679, C641, C642, C649, C651, C652, C659, C661, C662, C669, C680, C681, C688, C689 |
| Hematologic cancer | C8100, C8101, C8102, C8103, C8104, C8105, C8106, C8107, C8108, C8109, C8110, C8111, C8112, C8113, C8114, C8115, C8116, C8117, C8118, C8119, C8120, C8121, C8122, C8123, C8124, C8125, C8126, C8127, C8128, C8129, C8130, C8131, C8132, C8133, C8134, C8135, C8136, C8137, C8138, C8139, C8140, C8141, C8142, C8143, C8144, C8145, C8146, C8147, C8148, C8149, C8170, C8171, C8172, C8173, C8174, C8175, C8176, C8177, C8178, C8179, C8190, C8191, C8192, C8193, C8194, C8195, C8196, C8197, C8198, C8199, C8200, C8201, C8202, C8203, C8204, C8205, C8206, C8207, C8208, C8209, C8210, C8211, C8212, C8213, C8214, C8215, C8216, C8217, C8218, C8219, C8220, C8221, C8222, C8223, C8224, C8225, C8226, C8227, C8228, C8229, C8230, C8231, C8232, C8233, C8234, C8235, C8236, C8237, C8238, C8239, C8240, C8241, C8242, C8243, C8244, C8245, C8246, C8247, C8248, C8249, C8250, C8251, C8252, C8253, C8254, C8255, C8256, C8257, C8258, C8259, C8260, C8261, C8262, C8263, C8264, C8265, C8266, C8267, C8268, C8269, C8280, C8281, C8282, C8283, C8284, C8285, C8286, C8287, C8288, C8289, C8290, C8291, C8292, C8293, C8294, C8295, C8296, C8297, C8298, C8299, C8300, C8301, C8302, C8303, C8304, C8305, C8306, C8307, C8308, C8309, C8310, C8311, C8312, C8313, C8314, C8315, C8316, C8317, C8318, C8319, C8330, C8331, C8332, C8333, C8334, C8335, C8336, C8337, C8338, C8339, C8350, C8351, C8352, C8353, C8354, C8355, C8356, C8357, C8358, C8359, C8370, C8371, C8372, C8373, C8374, C8375, C8376, C8377, C8378, C8379, C8380, C8381, C8382, C8383, C8384, C8385, C8386, C8387, C8388, C8389, C8390, C8391, C8392, C8393, C8394, C8395, C8396, C8397, C8398, C8399, C8400, C8401, C8402, C8403, C8404, C8405, C8406, C8407, C8408, C8409, C8410, C8411, C8412, C8413, C8414, C8415, C8416, C8417, C8418, C8419, C8440, C8441, C8442, C8443, C8444, C8445, C8446, C8447, C8448, C8449, C8460, C8461, C8462, C8463, C8464, C8465, C8466, C8467, C8468, C8469, C8470, C8471, C8472, C8473, C8474, C8475, C8476, C8477, C8478, C8479, C8490, C8491, C8492, C8493, C8494, C8495, C8496, C8497, C8498, C8499, C84A0, C84A1, C84A2, C84A3, C84A4, C84A5, C84A6, C84A7, C84A8, C84A9, C84Z0, C84Z1, C84Z2, C84Z3, C84Z4, C84Z5, C84Z6, C84Z7, C84Z8, C84Z9, C8510, C8511, C8512, C8513, C8514, C8515, C8516, C8517, C8518, C8519, C8520, C8521, C8522, C8523, C8524, C8525, C8526, C8527, C8528, C8529, C8580, C8581, C8582, C8583, C8584, C8585, C8586, C8587, C8588, C8589, C8590, C8591, C8592, C8593, C8594, C8595, C8596, C8597, C8598, C8599, C860, C861, C862, C863, C864, C865, C866, C880, C882, C883, C884, C888, C889, C9000, C9001, C9002, C9010, C9011, C9012, C9020, C9021, C9022, C9030, C9031, C9032, C9100, C9101, C9102, C9110, C9111, C9112, C9130, C9131, C9132, C9140, C9142, C9150, C9151, C9152, C9160, C9161, C9162, C9190, C9191, C9192, C91A0, C91A1, C91A2, C91Z0, C91Z1, C91Z2, C9200, C9201, C9202, C9210, C9211, C9212, C9220, C9221, C9222, C9230, C9231, C9232, C9240, C9241, C9242, C9250, C9251, C9260, C9261, C9262, C9290, C9291, C9292, C92A0, C92A1, C92Z0, C92Z1, C92Z2, C9300, C9301, C9302, C9310, C9311, C9312, C9330, C9331, C9332, C9390, C9391, C9392, C93Z0, C93Z1, C93Z2, C9400, C9401, C9402, C9420, C9421, C9422, C9430, C9431, C9432, C9440, C9401, C9402, C9420, C9421, C9422, C9430, C9431, C9432, C9440, C9441, C9442, C946, C9480, C9481, C9482, C9500, C9501, C9502, C9510, C9511, C9512, C9590, C9591, C9592, C960, C962, C9620, C9621, C9622, C9629, C964, C965, C966, C969, C96A, C96Z |
| Other cancers | C33, C3400, C3401, C3402, C3410, C3411, C3412, C342, C3430, C3431, C3432, C3480, C3481, C3482, C3490, C3491, 3492, C384, C390, C399, C4000, C4001, C4002, C4010, C4011, C4012, C4020, C4021, C4022, C4030, C4031, C4032, C4080, C4081, C4082, C4090, C4091, C4092, C410, C411, C412, C413, C414, C419, C4A0, C4A10, C4A11, C4A111, C4A112, C4A12, C4A121, C4A122, C4120, C4A21, C4A22, C4A30 C4A31, C4A39, C4A4, C4A51, C4A52, C4A59, C4A60, C4A61, C4A62, C4A70, C4A71, C4A72, C4A8, C4A9, C430, C4310, C4311, C43111, C43112, C4312, C43121, C43122, C4320, C4321, C4322, C4330, C4331, C4339, C434, C4351, C4352, C4359, C4360, C4361, C4362, C4370, C4371, C4372, C438, C439, C4400, C4401, C4402, C4409, C44101, C44102, C441022, C44109, C441091, C441092, C44111, C44112, C441121, C441122, C44119, C441191, C441192, C44121, C44122, C441221, C441222, C44129, C441291, C441292, CC44131, C441321, C441322, C441391, C441392, C44191, C44192, C441921, C441922, C44199, C441991, C441992, C44201, C44202, C44209, C44211, C44212, C44219, C44221, C44222, C44229, C44291, C44292, C44299, C44300, C44301, C44309, C44310, C44311, C44319, C44320, C44321, C44329, C44390, C44391, C44399, C4440, C4441, C4442, C4449, C44500, C44501, C44509, C44510, C44511, C44519, C44520, C44521, C44529, C44590, C44591, C44599, C44602, C44609, C44611, C44612, C44622, C44629, C44691, C44692, C44699, C44701, C44702, C44709, C44711, C44719, C44721, C44722, C44729, C44791, C44792, C44799, C4480, C4481, C4482, C4489, C4490, C4491, C4492, C4499, C50011, C50012, C50019, C50021, C50022, C50029, C50111, C50112, C50122, C50129, C50211, C50212, C50219, C50221, C50222, C50229, C50311, C50312, C50319, C50321, C50322, C50329, C50411, C50412, C50419, C50421, C50422, C50429, C50511, C50512, C50519, C50521, C50522, C50529, C50611, C50612, C50619, C50621, C50622, C50629, C50811, C50812, C50819, C50821, C50822, C50829, C50911, C50912, C50919, C50921, C50922, C50929, C6900，C6901, C6902, C6910, C6911, C6912, C6920, C6921, C6922, C6930, C6931, C6932, C6940, C6941, C6942, C6950, C6951, C6952, C6960, C6961, C6962, C6980, C6981, C6982, C6990, C6991, C6992, C700, C709, C710, C711, C712, C713, C714, C715, C716, C717, C718, C719, C720, C721, C7220, C7221, C7222, C7230, C7231, C7232, C7240, C7241, C7242, C7250, C7259, C729, C7A0, C7A010, C7A011, C7A012, C7A019, C7A020, C7A021, C7A022, C7A023, C7A024, C7A025, C7A026, C7A029, C7A090, C7A091, C7A092, C7A093, C7A094, C7A095, C7A096, C7A098, C784, C785, C786, C787, C7880, C7889, C770, C771, C772, C773, C774, C775, C778, C779, C73, C7400, C7401, C7402, C7410, C7411, C7412, C7490, C7491, C7492, C750, C751, C752, C753, C754, C755, C758, C759, C760, C761, C762, C763, C7640, C7641, C7642, C7650, C7651, C7652, C768, C490, C4910, C4911, C4922, C493, C494, C495, C496, C498, C499, C450, C451, C452, C457, C459, C460, C461, C462, C463, C464, C4650, C4651, C4652, C467, C469, C380, C381, C382, C383, C388, C784, C785, C786, C787, C7880, C7889, C7800, C7801, C7802, C781, C782, C7830, C7839, C7900, C7901, C7902, C7910, C7911, C7919, C792, C7931, C7932, C7940, C7949, C7951, C7952, C7970, C7971, C7972, C7981, C7989, C799, C7960, C7961, C7962, C7982, C792, C7931, C7932, C7940, C7949, C7951, C7952, C7970, C7971, C7972, C7981, C7989, C799 |
| Diagnostic peritoneal paracentesis | 0W9G0ZX, 0W9G3ZX, 0W9G4ZX |
| Peritoneal drainage | 0W9G3ZZ, 0W9G4ZZ |
| Peritoneal drainage with drainage devices | 0W9G30Z, 0W9G40Z |
| Obesity | E6601, E6609, E661, E662, E663, E668, E669 |
| Hypertension | I10, I110, I119, I120, I129, I130, I1310, I1311, I132 |
| Hyperlipemia | E780, E7800, E7801, E781, E782, E783, E784, E7841, E7849, E785 |
| Diabetes | E0800, E0801, E0810, E0811, E0821, E0822, E0829, E08311, E08319, E08321, E083211-E083213, E083219, E08329, E083291-E083293, E083299, E08331, E083311-E083313, E083319, E08339, E083391-E083393, E083399, E08341, E083411-E083413, E083419, E08349, E083491-E083493, E083499, E08351, E083511-E083513, E083519, E083521-E083523, E083529, E083531-E083533, E083539, E083541-E083543, E083549, E083551-E083553, E083559, E08359, E083591-E083593, E083599, E0836, E0837X1-E0837X3, E0837X9, E0839-E0844, E0849-E0852, E0859, E08610, E08618, E08620, E08621, E08622, E08628, E08630, E08638, E08641, E08649, E0865, E0869, E088, E089, E0900, E0901, E0910, E0911, E0921, E0922, E0929, E09311, E09319, E09321, E093211-E093213, E093219, E09329, E093291-E093293, E093299, E09331, E093311-E093313, E093319, E09339, E093391-E093393, E093399, E09341, E093411-E093413, E093419, E09349, E093491-E093493, E093499, E09351, E093511-E093513, E093519, E093521-E093523, E093529, E093531-E093533, E093539, E093541-E093543, E093549, E093551-E093553, E093559, E09359, E093591-E093593, E093599, E0936, E0937X1-E0937X3, E0937X9, E0939-E0944, E0949, E0951, E0952, E0959, E09610, E09618, E09620-E09622, E09628, E09630, E09638, E09641, E09649, E0965, E0969, E098, E099, E101, E1011, E1021, E1022, E1029, E10311, E10319, E10321, E103211-E103213, E103219, E10329, E103291-E103293, E103299, E10341, E103411-E103413, E103419, E10349, E103491-E103493, E103499, E10351, E103511-E103513, E103519, E103521-E103523, E103529, E103531-E103533, E103539, E103541-E103543, E103549, E103551-E103553, E103559, E10359, E103591-E103593, E103599, E1036, E1037X1-E1037X3, E1037X9, E1039-E1044, E1049, E1051, E1052, E1059, E10610, E10618, E10620, E10621, E10622, E10628, E10630, E10638, E10641, E10649, E1065, E1069, E108, E109, E1100, E1101, E1110, E1111, E1121, E1122, E1129, E11311, E11319, E11321, E113211-E113213, E113219, E11329, E113291-E113293, E113299, E11331, E113311-E113313, E113319, E11339, E113391-E113393, E113399, E11341, E113411-E113413, E113419, E11349, E113491-E113493, E113499, E11351, E113511-E113513, E113519, E113521-E113523, E113529, E113531-E113533, E113539, E113541-E113543, E113549, E113551-E113553, E113559, E11359, E113591-E113593, E113599, E1136, E1137X1-E1137X3, E1137X9, E1139-E1144, E1151, E1152, E1159, E11610, E11618, E11620-E11622, E11628, E11630, E11638, E11641, E11649, E1165, E1169, E118, E119, E1300, E1301, E1310, E1311, E1321, E1322, E1329, E13311, E13319, E13321, E133211-E133213, E133219, E13329, E133291-E133293, E133299, E13331, E133311-E133313, E133319, E13339, E133391-E133393, E133399, E13341, E133411-E133413, E133419, E13349, E133491-E133493, E133499, E13351, E133511-E133513, E133519, E133521-E133523, E133529, E133531-E133533, E133539, E133541-E133543, E133549, E133551-E133553, E133559, E13359, E133591-E133593, E133599, E1336, E1337X1-E1337X3, E1337X9, E1339-E1344, E1349, E1351, E1352, E1359, E13610, E13618, E13620-E13622, E13628, E13630, E13638, E13641, E13649, E1365, E1369, E139 |
| Chronic kidney disease | N181, N182, N183, N1830, N1831, N1832, N184, N185, N186, N189 |
| Heart failure | I1501, I15020, I15021, I15022, I15023, I15030, I15031, I15032, I15033, I15040, I15041, I15042, I15043, I150811, I150812, I150813, I150814, 15082, I5083, I5084, I5089, I509 |
